# Catching Cold

**Published:** 1874-06

**Authors:** 


*DETROIT REVIEW OF MEDICINE AND PHARMACY—February,* 1874:

EFFECTS OF SUDDEN CHANGES OF TEMPERATURE IN "CATCHING COLD."—In the *British Medical Journal,* June 28, 1873, is given an account of Prof. Rosenthal's researches upon the above subject. It has long been known that lowness of temperature did not produce colds, but rather sudden changes from a higher to a lower. The application of cold to the surface of a healthy animal causes the cutaneous vessels to contract. Thus they prevent the blood from circulating in the skin, and by confining it to the interior of the body, prevent its cooling and preserve the temperature of the vital organs, unless the application be continued for a long time. But if the animal be exposed to heat, the cutaneous vessels become paralyzed, and remain dilated after exposure to cold. The blood is thus exposed in large amount over a large surface, and becomes rapidly cooled, even although the temperature of the surrounding medium is not very low. As people pass from a choky office, hot theater, or crowded ball-room, into a cool draught of air, the blood, which is coursing not only over the flushed face, but through the dilated vessels of every part of the surface, is rapidly cooled below the normal. Returning to the internal organs, it cools them much more quickly than it could have done had the person been exposed to cold without dilatation of vessels by previous warmth. Thus a *sudden* cooling of the blood produces an irritating effect, or induces an inflammation in a weak organ, in a way that a *gradual* alteration would not do. To produce evil results the cooling must be from *above* to *below* the normal temperature. The effect of a chill in causing inflammation may be partly due to the effect of cold on the tissues themselves, and partly to the hyperæmia which will occur in some parts when the blood is driven out of others by the contraction of vessels. Rosenthal lays most importance on the first of these effects. It is a well known fact that frequent cold bathing or sponging enables one to bear, without harm, sudden changes of weather. This is explained by an increased tone in the vessels, produced by the cold applications. Thus when exposed to heat they are not so relaxed that they cannot sufficiently contract when necessary. Thus the power of regulating the temperature is relaxed, and catching cold prevented.

